# “Leaving no one behind” also includes taking the elderly along concerning their sexual and reproductive health and rights: a new focus for *Reproductive Health*

**DOI:** 10.1186/s12978-020-00944-5

**Published:** 2020-06-29

**Authors:** Aduragbemi Banke-Thomas, Comfort Z. Olorunsaiye, Sanni Yaya

**Affiliations:** 1 LSE Health, London School of Economics and Political Science, London, UK; 2 Department of Public Health, Arcadia University, Glenside, PA USA; 3 The George Institute for Global Health, The University of Oxford, Oxford, UK; 4 School of International Development and Global Studies, Faculty of Social Sciences, University of Ottawa, Ottawa, Canada

It is now well established that the world’s population is ageing, and has been doing so rapidly in the last century. According to the United Nations, as of 1950, there were an estimated 205 million people aged 60 years or over living in the world. More recently, that number had almost quintupled, with the 2017 estimate put at 962 million [1, 2]. Two-thirds of the older population aged 60 years or over live in low- and middle-income countries, where their rate of growth has been faster compared to high-income countries. Put together, the number of people aged 60 years or older is expected to keep increasing globally, with a further doubling projected by the year 2050, at 2.1 billion, by which point this elderly cohort would have outstripped adolescents, who are expected to number 2.0 billion [1].

Ageing will have a significant impact on health and social policies, and the older people (both men and women) will likely be among the policy-making priorities in the next few decades. Indeed, there have been several areas of health of the elderly that are increasingly receiving attention including physical health, primarily focused on non-communicable diseases, and mental health, mainly dementia and depression [3, 4]. However, sexual and reproductive health and rights (SRHR) issues in older adults remain a “taboo” among individuals and in many societies at large, a “topic of minimal interest” for health professionals and researchers; and a “blind spot” in the broader policy dialogue [4, 5]. This minimal focus remains despite the global call for the need to implement a lifecourse approach, from pre-pregnancy to post-reproductive years, in tackling SRHR issues [6, 7].

There are undoubtedly several SRHR issues relevant to the older population. For older women, a sharp decline in oestrogen and progesterone levels leads to declining ovarian function and then to several physical and psychological changes experienced as part of the peri-menopausal syndrome, including reduced libido, lack of energy, osteoporosis, irritability and mood swings. In some cultures, women perceive menopause as a positive experience, choosing to focus more on their freedom from menstruation; others see menopause as a phenomenon requiring medical intervention [8, 9]. Older men, on the other hand, have reductions in their testosterone levels and sperm production gradually becomes lower. While they also experience some similar physical and psychological symptoms, as a result of these low levels, there are a few differences including a decrease in lean body mass, decrease in body hair, skin alterations, erectile dysfunction, and increase in visceral fat and obesity. Besides, the development of symptoms is a lot more gradual compared to menopause that women experience [10]. For both men and women, there is also the impact of non-communicable diseases and medications that older people typically take, and their impact on libido, erectile dysfunction and energy levels. Non-communicable diseases at older age are exacerbated in women as a result of the cumulative impact of pregnancy and childbearing during the reproductive age.

For older people, there is a general perception that they do not have any sexual desires and even if they do, in many cultures, they are not expected to speak about such matters [11]. However, available evidence suggests that more than 80% of men and 65% of women remain sexually active in old age, with 28 and 39% of older men and women, respectively, reporting that they are affected by at least one sexual dysfunction [12]. Of course, what this sustained sexual activity into old age means is that there remains a risk for sexually transmitted infections (STIs) and Human immuno-Deficiency Virus (HIV) infection amongst the older population [13]. For older women, in particular, the increased vaginal dryness, atrophy of the vaginal wall and loss of lubrication which they experience as part of menopause, interfere with sexual comfort and pleasure. These symptoms also limit the effectiveness of innate vaginal protective mechanisms against STIs and HIV infection. The minimal perception of risk for STIs amongst older persons is another contributory factor, as they do not tend to use contraception during sexual activity since they do not need this for birth control. However, collaterally older people do not then have any protection from STIs that they may be exposed to when they engage in sexual activity. For widows and widowers who have no partners, they also have desires for sensual interactions consisting of nonsexual intimacy as opposed to sexual experiences [14].

When older people choose to engage with the health system to discuss and seek help regarding their SRHR needs, it becomes inherently clear that the health systems are not designed to meet their needs, or address their issues. In many settings, health workers have been described as being stereotypical, prejudiced and discriminatory against older people based on their age. A 2017 World Health Organization study estimated that, in 2016, about 16% of people aged 60 years and older had been subjected to some form of abuse including sexual abuse, typically committed by people in a position of trust including health care providers and family members [15]. Moreover, even when data on the adequacy of service provision needs to be explored, it is not easy to find data on SRHR of older populations, as they are not systematically collected [16].

In the past three decades, efforts have been made to draw the much needed attention to this critical issue of SRHR of the older population. Firstly, around the launch of the Millennium Development Goals [8, 17] and more recently around the International Conference on Population and Development’s Beyond 2014: International Conference on Human Rights, older people were explicitly recognised as one of four key population groups that have been marginalised and excluded in their access to SRH and rights [5, 13]. However, these calls have not yielded any significant number of research articles in the subject area, more so in low- and middle-income countries, where even fewer studies have been conducted [13]. The Sustainable Development Goal (SDG) 3, which is aimed at ensuring healthy lives and promoting wellbeing for all at all ages [18], offers a unique opportunity to place more emphasis on capturing the views of older people while promoting more age-inclusive health systems and services that guarantee an improved quality of life. In the World report on ageing and health published in 2015, healthy ageing is defined as “the process of developing and maintaining the functional ability that enables wellbeing in older age” [19]. This definition was re-emphasised as this decade (2020–2030) was declared as the decade of healthy ageing [20]. However, to realise true healthy ageing, SRH and rights issues cannot be ignored.

To create a platform for robust discussions, the *Reproductive Health* journal is launching a new and dedicated section called “Elderly Reproductive Health” and now welcomes submissions that would help better understand the needs and issues related to the reproductive health of this age group. Beyond the need to understand SRHR issues of older people in various settings and cultures, there is also a need to understand the best approaches for implementation, effectiveness and cost-effectiveness of interventions that can help improve experiences, outcomes and quality of life of older persons. Furthermore, there is a case for cross-national research that will allow for comparisons and shared lessons across borders. With the SDG vision of leaving no one behind, we hope that this newly launched platform makes it more ‘sexy’ to share about elderly sexual and reproductive health.
